# The need for standardization and improved open (meta)data practices in metaproteomics

**DOI:** 10.1186/s40168-026-02455-0

**Published:** 2026-07-23

**Authors:** Tim Van Den Bossche, Maximilian Wolf, Jean Armengaud, Magnus Ø. Arntzen, Dirk Benndorf, Daniel Figeys, Lucia Grenga, Robert L. Hettich, Nithu Sara John, Pratik Jagtap, Nico Jehmlich, Manuel Kleiner, Benoit J. Kunath, Leyuan Li, Mary Lipton, Bart Mesuere, Benjamin A. Neely, Zhibin Ning, Ane Laura Pedersen, Yasset Perez-Riverol, Jeena Rajan, Kay Schallert, Jana Seifert, Sergio Uzzau, Pieter Verschaffelt, Paul Wilmes, Juan Antonio Vizcaíno, Lennart Martens, Robert Heyer

**Affiliations:** 1 CompOmics, VIB-UGent Center for Medical Biotechnology, Ghent, Belgium; 2 Department of Biomolecular Medicine, Faculty of Medicine and Health Sciences, Ghent University, Ghent, Belgium; 3 Multidimensional Omics Analyses Group, Faculty of Technology, Bielefeld University, Bielefeld, Germany; 4 Graduate School DILS, Bielefeld Institute for Bioinformatics Infrastructure (BIBI), Bielefeld University, Bielefeld, Germany; 5 Département Médicaments Et Technologies Pour La Santé (DMTS), SPI, Université Paris-Saclay, CEA, INRAE, Bagnols-Sur-Cèze, France; 6 Faculty of Chemistry, Biotechnology and Food Science, Norwegian University of Life Sciences, Ås, Norway; 7 Applied Biosciences and Process Engineering, Anhalt University of Applied Science, Köthen, Germany; 8 Faculty of Medicine, University of Ottawa, Ottawa, Canada; 9 Biosciences Division, Oak Ridge National Laboratory, Oak Ridge, USA; 10 Wellcome Trust Genome Campus, European Bioinformatics Institute, Cambridge, UK; 11 Department of Biochemistry, Molecular Biology, and Biophysics, Minneapolis, USA; 12 Department of Molecular Toxicology, Helmholtz Centre for Environmental Research GmbH - UFZ, Leipzig, Germany; 13 Department of Plant and Microbial Biology, North Carolina State University, Raleigh, USA; 14 Department of Cancer Research, Multiomics Data Science Research Group, Luxembourg Institute of Health, Strassen, Luxembourg; 15 State Key Laboratory of Medical Proteomics, Beijing Proteome Research Center, Beijing, China; 16 Earth and Biological Sciences Directorate, Pacific Northwest National Laboratory, Richland, USA; 17 Department of Mathematics, Computer Science and Statistics, Ghent University, Ghent, Belgium; 18 National Institute of Standards and Technology, Charleston, USA; 19 Nestlé Institute of Food Safety & Analytical Sciences, Nestlé Research, Lausanne, Switzerland; 20 Leibniz-Institut für Analytische Wissenschaften–ISAS–e.V., Dortmund, Germany; 21 HoLMiR—Hohenheim Center for Livestock Microbiome Research, University of Hohenheim, Stuttgart, Germany; 22 Department of Biomedical Sciences, University of Sassari, Sassari, Italy; 23 Luxembourg Centre for Systems Biomedicine, University of Luxembourg, Luxembourg, Luxembourg

## Abstract

Metaproteomics enables functional insight into microbial communities by identifying and quantifying proteins in complex samples. Yet, heterogeneous analytical workflows and the lack of standardization across experimental and bioinformatics stages hinder reproducibility and comparability, limiting integration with other omics data. We here present a community-developed reporting checklist tailored to the specific needs of metaproteomics. We also outline current efforts to enable structured and interoperable metadata capture, drawing on standards from proteomics and microbiome research wherever possible. By promoting transparent reporting and advancing metadata practices, our recommendations aim to align metaproteomics more closely with FAIR principles and support reproducible and interoperable research practices.

Video Abstract

**Supplementary Information:**

The online version contains supplementary material available at 10.1186/s40168-026-02455-0.

## Introduction

Metaproteomics, the large-scale characterization of proteins from complex microbial communities, provides a direct view of microbial function by identifying and quantifying protein abundance at specific sampling conditions. Metaproteomics complements the other related omics approaches by providing detailed information about context-dependent, dynamic proteome expression, including information about protein post-translational modifications. As the field matures, metaproteomics is being increasingly applied to clinical, environmental, and industrial microbiome studies to investigate microbial phenotypes, host interactions, and functional dynamics, among other topics [1].

As an emerging technology, metaproteomics comprises a diversity of experimental and bioinformatics workflows, with protocol choices often tailored to sample type and instrumentation. Standardized reporting practices remain limited, which is not unexpected in a rapidly evolving discipline. Improved standardization would enhance the reuse of public datasets, increase transparency in study design, and support the development of scalable, interoperable infrastructure for multi-omics integration. This, in turn, would lower barriers for newcomers entering the field, thus ushering fresh talent, diverse perspectives, and innovation [2].

Several early papers helped raise awareness around the need for better reporting in metaproteomics. In 2019, Zhang and Figeys [3] provided a perspective on reporting guidelines, and Saito et al*.* proposed concrete recommendations for data and metadata sharing in ocean metaproteomics [4]. These papers laid important conceptual foundations and catalyzed broader community discussions. In addition, the Critical Assessment of Metaproteome Investigation (CAMPI) study [5] emerged as the first community-driven, multi-laboratory benchmark study coordinated by the Metaproteomics Initiative [1, 6]. CAMPI demonstrated the importance of consistent reporting and highlighted the need for standardization across experimental and computational workflows.

Building on these efforts, the Metaproteomics Initiative has since coordinated discussions on which experimental and computational parameters should be reported, how they might be structured, and to what extent existing standards can be reused or need adaptation. This work represents the first concrete outcome of those discussions, which are aligned with the FAIR (Findable, Accessible, Interoperable, and Reusable) data principles [7].

**Fig. 1:**
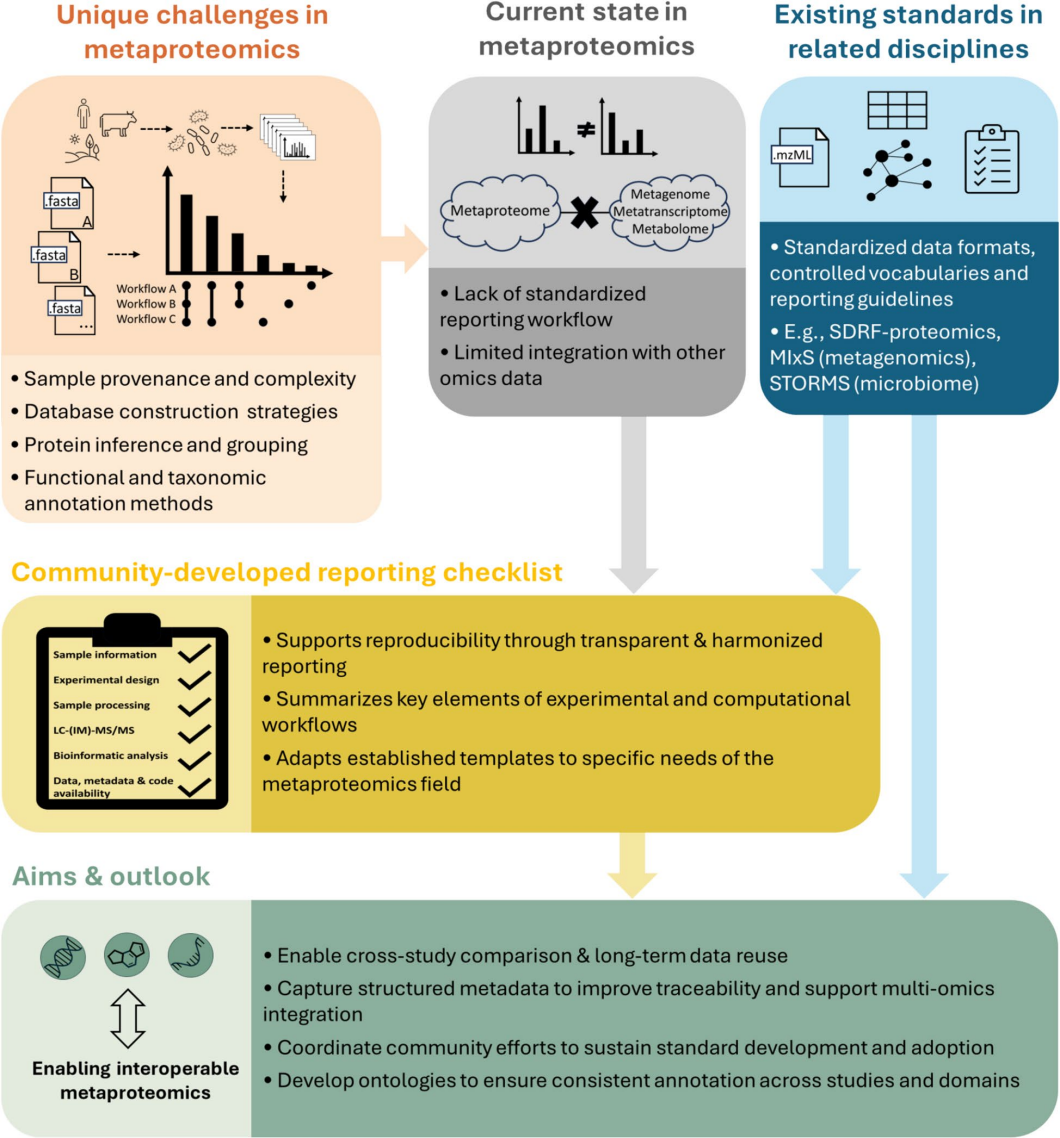
Overview of the current state and future directions in metaproteomics standardization

We here discuss current challenges in metaproteomics reporting and explore how the field can adopt standards from related disciplines, such as single-organism proteomics and metagenomics, while also addressing its distinct requirements (Fig. 1). We highlight which existing metadata standards can be reused and where tailored solutions are needed, and present a practical reporting checklist alongside ongoing efforts to develop structured metadata templates suited to metaproteomics. Our guidance focuses on both metadata and analysis parameters that are critical for ensuring reproducibility, interpretability, and cross-study integration, including sample origin, preservation methods, acquisition settings, database construction, and protein inference strategies. While recent work [8] has outlined broader challenges and opportunities for microbiome data sharing, this Perspective complements those efforts by offering domain-specific recommendations tailored to the metaproteomics community.

## Why does metaproteomics pose unique challenges?

Metaproteomics builds on principles and tools from proteomics and other meta-omics approaches, but faces distinct analytical challenges requiring dedicated solutions.

A fundamental challenge in metaproteomics is the documentation of sample provenance. Environmental and host-associated samples are often collected under diverse conditions and may represent complex or poorly defined microbial communities. Without structured information on sample origin, context, and collection procedures, data interpretation becomes ambiguous and reproducibility suffers. Moreover, provenance metadata are rarely integrated into public datasets, making it difficult to relate functional protein patterns back to ecological or clinical context.

A central issue is sample composition. In contrast to single-species proteomics, metaproteomics involves proteins from a diverse set of organisms, many of which are closely related, leading to conserved peptides with high sequence similarity across taxa. This reduces taxonomic resolution and complicates peptide-to-taxon assignment. To address this, Zhang and Figeys proposed a distinction between unique peptides, which are unique to a protein group but may still occur in proteins from multiple microbes, and distinctive peptides, which match only a single taxonomic group (e.g., a species or genus). Distinctive peptides may therefore represent a subset of unique peptides but can also include peptides shared between different proteins within the same taxonomic group. Peptides that are distinctive at lower taxonomic levels remain distinctive at higher levels, whereas the reverse is not necessarily true [3]. For example, a peptide that occurs in homologous proteins from two species within the same genus may be unique to a protein group but not distinctive at the species level. However, if it occurs only within that genus, it remains distinctive at the genus level. It is important to note that peptide uniqueness is always database-dependent: a peptide considered unique or taxonomically distinctive in one database may occur in organisms absent from that database. While metagenomic sequencing also deals with complex microbial samples, it achieves higher taxonomic resolution than metaproteomics since sequencing long contigs or metagenome-assembled genomes (MAGs) provides more taxonomically informative markers than the assortment of short peptides.

Additional analytical limitations stem from sample complexity. The dynamic range of microbial proteins typically exceeds the detection capabilities of current mass spectrometers, biasing identification toward more abundant proteins and taxa. Moreover, matrix effects from complex environmental samples (e.g., stool, soil, or marine samples) often require tailored protein extraction and cleanup protocols to achieve adequate protein recovery. These factors are rarely standardized and are often underreported, despite their known impact on downstream results [9].

Challenges also arise from how protein sequence databases are constructed. Databases may be derived from sample-specific metagenomes, curated reference genomes, or broad public repositories, but criteria for selecting, filtering, and combining sequences are rarely standardized. While ProteomeXchange repositories allow the submission of FASTA files, these are not consistently shared or sufficiently described. Unlike metagenomics, which benefits from established workflows for sharing reads, assemblies, and annotations, metaproteomics currently lacks comparable guidance for database reporting.

Protein inference in metaproteomics is inherently more ambiguous than in single-species proteomics [10] due to the complex sample composition described above. Since many proteins are inferred from a single peptide, and many peptides are shared across homologous proteins from different organisms, protein grouping or subgrouping becomes necessary, which complicates interpretation and reduces comparability across studies. These differences also affect how identification metrics are reported: the number of proteins can vary substantially depending on whether protein groups or individual proteins are counted, and on the inference strategy applied. Additionally, while both peptide-centric and protein-centric inference methods are used for taxonomic annotation, they are based on different assumptions and can lead to divergent taxonomic profiles.

Most environmental microbial proteins remain poorly annotated, and functional inference often depends on homology to proteins from model organisms. As a result, functional profiles are often incomplete and shaped by the structure and coverage of the underlying databases. Although these limitations are not exclusive to metaproteomics, they are especially pronounced given the diversity and novelty of environmental sequences.

Taken together, these challenges differentiate metaproteomics from both single-species proteomics and other meta-omics approaches. They underscore the need for dedicated guidelines encompassing experimental design, database construction, protein inference, and downstream analysis. Although several reporting elements overlap with general proteomics, they become particularly important in metaproteomics because database composition strongly affects identifications, shared peptides complicate taxonomic assignment, and downstream interpretation depends on both taxonomic and functional annotation. Aligning these guidelines with existing standards from related fields will improve interoperability and promote long-term reuse of metaproteomics data.

## Learning from other fields: what we can and can’t reuse

Standardization efforts in proteomics and microbiome research have resulted in widely adopted data formats, controlled vocabularies, and reporting guidelines. These offer a valuable foundation for metaproteomics, but they do not address all of its specific challenges. While some principles and tools can be reused directly, others require adaptation to accommodate the complexity of metaproteomics data. To identify which existing frameworks may be reused or adapted, we review relevant standards from proteomics and metagenomics below.

In proteomics, the Human Proteome Organization Proteomics Standards Initiative (HUPO-PSI) [11] has developed widely adopted frameworks for data sharing and standardization, including mzML [12] for raw mass spectrometry data, mzIdentML [13] for identification results, and the Universal Spectrum Identifier (USI) [14] for referencing individual spectra. Earlier efforts, such as MIAPE [15], outlined minimum reporting requirements for proteomics experiments, while journal-led standards (e.g., those from *Molecular & Cellular Proteomics*) further supported methodological transparency. More recently, the Sample and Data Relationship Format for Proteomics (SDRF-Proteomics) [16] is a structured tab-delimited metadata format that describes the relationships between samples and mass spectrometry data files. In this format, each row represents the link between a sample and a data file, while the columns capture sample characteristics and experimental design information. While these standards provide a strong foundation, they were originally developed for single-organism (human) proteomics and do not yet support many metadata fields relevant to microbial communities. Related community-driven efforts in metabolomics [17] and lipidomics [18] have also shown how structured templates can improve transparency, reproducibility, and data reuse. However, these standards were originally developed for single-organism studies and do not yet cover many metadata fields relevant to microbial ecosystems.

From the genomics field, the Minimum Information about any (x) Sequence (MIxS) framework [19] developed by the Genomics Standards Consortium (GSC) defines core metadata fields for sequencing experiments. It includes environmental extensions that cover biome type, substrate, pH, and host-associated parameters. Though developed for metagenomics, many of these descriptors are also relevant to metaproteomics. Additionally, earlier initiatives, such as the International Human Microbiome Standards (IHMS) project, focused on upstream workflows, such as sample collection, DNA extraction, and metadata for sequencing, but they did not address metaproteomics-specific requirements.

Related initiatives in microbiome research offer further inspiration. The STORMS checklist [20], developed for human microbiome studies, and STREAMS [21], for non-human, environmental, and synthetic microbiome studies, provide manuscript-aligned templates that emphasize transparency in metadata, sample handling, and statistical reporting.

Cross-domain initiatives are also actively contributing to metadata harmonization. The RDA Multi-Omics Metadata Standards Integration (MOMSI) working group has cataloged over 240 standards across different omics domains, distinguishing between general-purpose and domain-specific frameworks [22]. The ELIXIR Microbiome Community [23] is working on the sustainability and usability of bioinformatics resources, with a focus on supporting interoperability across technologies, ecosystems, and multi-omics platforms. Importantly, both MOMSI and ELIXIR explicitly include (meta)proteomics in their scope.

Together, these efforts offer a strong foundation for metaproteomics standardization. Nevertheless, critical metadata elements remain unsupported or insufficiently developed. These include strategies for protein grouping, methods for peptide-to-taxon assignment, and the provenance of functional annotations, all of which are essential for ensuring reproducibility and interpretability in metaproteomics studies.

## Recommended reporting practices in metaproteomics

A metaproteomics experiment typically consists of several stages: sampling of biological material, sample preparation and tandem mass spectrometry (MS/MS) data acquisition, bioinformatics processing, and downstream interpretation. Transparent reporting across these steps is essential to ensure reproducibility, facilitate comparisons across studies, and enable integration with other omics layers. A recent collective review under the umbrella of the Metaproteomics Initiative provides a comprehensive introduction to the complete workflow [24]*.* It is important to note that while several of these reporting elements are also relevant in general proteomics, they are particularly important in metaproteomics because complex microbial communities increase analytical ambiguity and make downstream taxonomic and functional interpretation more sensitive to experimental and computational choices.

At the experimental level, key contextual details about sampling, storage, and processing conditions are often underreported, yet they are critical for assessing comparability across datasets. Sample preparation protocols, including but not limited to cell lysis, protein extraction, digestion, peptide cleanup, and fractionation, should be described with sufficient detail to allow replication. Because metaproteomics often aims to detect low-abundance microbial signals and support taxonomic and functional interpretation, relevant measures taken to prevent, monitor, or account for contamination should be reported where applicable, e.g., through blank controls, digestion controls, carryover checks, or contaminant sequence handling during database searching, as exogenous proteins may influence downstream identifications and interpretation. Sample preservation is particularly important, as protein degradation, oxidation, and enzymatic activity can alter protein content [9]. Although standardized sampling protocols are increasingly used in microbiome studies, they often focus on nucleic acid preservation and are not optimized for protein stability. Storage temperature and duration have a documented impact: for example, degradation can occur at room temperature, while partial preservation is possible at − 20 °C or − 80 °C depending on the sample type [25]. Additionally, the CAMPI-2 study [9] of the Metaproteomics Initiative evaluated five preservation methods for fecal samples and showed that each method had trade-offs in terms of metaproteome integrity and reproducibility, affecting both taxonomic and functional profiles. This finding underscores the importance for detailed reporting of stabilization protocols. Where available, standard operating procedures (SOPs) should be referenced or made publicly accessible through platforms, such as protocols.io, to enhance transparency and accessibility. For clinical samples, ethical approvals, informed consent procedures, and data-sharing restrictions must be documented. For environmental samples, legal compliance with frameworks, such as the Nagoya Protocol, should be ensured. For MS analysis, details, such as instrument type, settings, and data acquisition strategies must be clearly reported, especially when using data-independent acquisition or ion mobility-enabled platforms.

Instrument quality control (QC) in metaproteomics should reflect the complexity and dynamic range of microbial communities. Reference materials are essential for assessing reproducibility and benchmarking analytical performance. Vertical reference materials (e.g., technical replicates, instrument standards) help monitor MS variability, while horizontal reference materials (designed to mimic the taxonomic and functional complexity of real samples) support evaluating pipeline performance, including database construction, protein inference, and taxonomic profiling. Additionally, experimental designs should include quality assurance practices such as sample randomization, injection order, and the use of pooled QC samples. The composition and preparation of all reference and control materials should be documented. Formats, such as mzQC [26], provide a structured framework for encoding instrument-level QC metrics.

Bioinformatics processing should specify the software tools used, their versions, database construction strategies, and parameters for search and validation. When custom or unpublished software is used, code and documentation should be made available through a repository, such as GitHub. Search parameters, threshold values, and post-processing steps (e.g., rescoring, FDR control, taxonomic annotation, functional mapping) must be explicitly described. All raw and processed data, including spectra, identification results, protein sequence databases (FASTA files), and metadata, should be deposited in ProteomeXchange repositories, such as PRIDE [27]. Although not all of these files are formally required by repository submission guidelines, their deposition is important to support transparency, reproducibility, and data reuse.

The bioinformatics processing of metaproteomics data includes peptide identification, protein inference and quantification, and transfer of taxonomic and functional annotations from the search database [28]. Because database composition directly affects peptide and protein identification, as well as downstream taxonomic and functional interpretation, deposition of the exact database used in ProteomeXchange is particularly important in metaproteomics. Reporting of database construction should include the sequence source(s), the number of entries, download date, and any applied filtering or preprocessing. For public databases, a full list of stable identifiers with version and download date is typically sufficient. In cases where suitable reference databases are unavailable or incomplete, de novo sequencing may be used to infer peptide sequences directly from MS/MS spectra, although this approach has known challenges and should be clearly documented [29].

Taxonomic annotation strategies must be described in detail, including the software used, version number, input types, filtering criteria, and quantification methods. Tools, such as Unipept [30] and Prophane [31], apply different strategies to assign peptides to taxa. It is essential to clarify whether assignments are based on distinctive peptides (defined as peptides observed only within a specific taxonomic group in the selected reference database) or include shared peptides. Since peptide uniqueness is database-dependent, as explained above, definitions and resolution thresholds must be explicitly stated. The achieved resolution (e.g., genus or species) and any post-processing (e.g., grouping of low-abundance taxa, reclassification) should also be reported.

Functional annotation requires equally clear reporting. Researchers should document the tools and versions used, assignment methods, similarity thresholds, and output summaries (e.g., protein counts per functional category). Given the limited coverage of current protein function databases, especially for non-model organisms, many studies rely on sequence-based prediction tools, such as InterProScan [32] or eggNOG [33], or metagenomic pipelines, such as PROKKA [34] and DRAM [35].

Reporting of downstream analysis should include parameters for missing value imputation, normalization, statistical analysis, and visualization. Missing value treatment must be clearly reported, as imputation can strongly influence biological interpretation. This is particularly important in metaproteomics, where missing values are more prevalent than in single-species proteomics due to lower coverage and sample complexity. Researchers must report the imputation algorithm used, its assumptions, and any filtering performed prior to imputation. If imputation-free statistical approaches are applied, their underlying assumptions and implications should be clearly explained.

To support transparent and harmonized reporting, we present a metaproteomics reporting checklist (Table 1). This checklist summarizes the key elements that should be documented across the experimental and computational workflow stages, including sampling, sample preservation, database construction, search settings, protein inference, and downstream analysis. Our checklist adapts well-established templates to the specific needs of metaproteomics, such as multi-organism samples, custom database strategies, and multi-omics integration. It is intended as a practical tool for researchers, reviewers, editors, and repositories, and can be downloaded from the Metaproteomics Initiative’s website (www.metaproteomics.org). While some elements can be encoded in structured formats, such as SDRF-Proteomics, others require narrative descriptions or protocol-level detail. The checklist should therefore be viewed as a flexible guide rather than a rigid template.

**Table 1:** Reporting checklist for metaproteomics experiments

Item to report	Recommended reporting
*Sample information*	
Sample source	Describe the origin of the sample (e.g., soil, human gut, marine), the surrounding environment, and relevant parameters.
Collection method	Report how the sample was collected, including the method used, sample volume or weight, and any pre-storage handling or processing.
Storage and preservation	Specify storage conditions (e.g., temperature, duration), preservation method, time between collection (removal of sample from environment) and preservation, and time kept in preservation until processing.
*Experimental design*	
Replicates	Report the number and type of replicates included in the study, clearly distinguishing between biological and technical replicates. Indicate how replicates were processed.
Statistical design	Describe the overall study design and statistical considerations, including randomization strategy, use of controls, blocking, and batch correction. If applicable, provide power calculations or a justification for the chosen number of replicates.
*Sample (pre-)processing*	
Pre-processing	Specify sample pre-processing steps, such as fractionation, e.g., by centrifugation or filtering, and concentration, e.g., with filters or lyophilization.
Cell lysis and homogenization	Specify the lysis method (e.g., mechanical, enzymatic, chemical), including any cell separation or inactivation steps. Report whether protease inhibitors were used.
Protein extraction	Describe the method used to extract proteins from lysed cells, including buffers or chemicals applied and any precipitation or cleanup steps.
Protein fractionation	Describe protein fractionation method (e.g., 1D-PAGE or chromatography).
Protein digestion	Detail the digestion protocol, including protein quantity, buffer composition, reduction and alkylation conditions, enzyme(s) used, incubation time and temperature, enzyme-to-protein ratio, and peptide cleanup method.
Peptide fractionation	Describe peptide-level prefractionation strategies applied prior to LC–MS/MS analysis (e.g., high-pH reversed-phase liquid chromatography, strong cation exchange).
Labelling	If chemical, metabolic or isotopic labeling was used (e.g., TMT, iTRAQ, Protein-SIP), describe the labeling protocol and any post-labeling processing steps.
Contamination control measures	Specify relevant measures used to prevent, monitor, or account for contamination, such as blank controls, digestion controls, or carryover checks, where applicable.
*LC-(IM)-MS/MS*	
LC separation conditions	Report LC parameters, including instrument, trap-and-elute vs. direct inject, column type, length, particle size, solvent composition, gradient types and elution conditions, flow rate, and peptide load.
Ion mobility separation conditions	If ion mobility was used, specify the technique (e.g., TIMS, TWIMS, FAIMS), drift gas type and pressure, temperature, electric field settings, and calibration method. If collision cross-section (CCS) values were reported, include the calibration procedure and associated uncertainty.
MS acquisition parameters	Specify the mass spectrometer model and configuration. Report acquisition mode (e.g., DDA or DIA), scan range, resolution, dynamic exclusion, and fragmentation settings. For DDA, include MS2 resolution and top N selection strategy; for DIA, specify isolation window size and scan strategy.
*Bioinformatics analysis*	
Protein database construction	Report the number of sequences in the search database and the download date if sourced from a public repository. If a custom database was used, describe in detail how it was generated, including the origin of the sequences, how taxa were selected or excluded, how redundancy was handled, and how both functional and taxonomic annotations were assigned. Indicate how contaminant sequences were incorporated.
Search engine and parameters	State which search engine has been used and which version number. Mention parameters that have been used in the search, including but not limited to precursor- and fragment ion tolerances, fixed (static) and variable (dynamic) modifications, number of allowed missed cleavages, and enzymatic specificity (e.g., tryptic, semi-tryptic).
FDR control	Define the false discovery rate (FDR) approach used and the thresholds applied at the peptide-spectrum match (PSM), and, where applicable, peptide, and protein or protein (sub)group levels. Specify whether global or local FDR was used.
Protein identification criteria	Specify the criteria used to report proteins or protein (sub)groups, including the minimum number of peptides required for identification and whether peptides must be unique or may include shared peptides.
Protein inference	Describe the algorithm or method used to group peptides into proteins and protein (sub)groups, including version and parameters where applicable.
Identification metrics	Report the number of PSMs, peptides, and proteins or protein (sub)groups identified at the defined FDR threshold.
Protein quantification	Indicate the quantification strategy applied (label-free or labeling), the normalization method, the proportion of missing values, and whether imputation was performed (including the method, if applicable).
Taxonomic profiling	Report the method or software used for taxonomic annotation, including version and relevant parameters. Provide the total number of taxonomic units identified (e.g., genera, species) and the number of peptides or proteins assigned to each. If taxonomic units were quantified, describe the quantification method. Include any filtering or binning steps (e.g., grouping low-abundance taxa) and justify these choices.
Functional annotation	Report the method or software used for functional annotation, including version and relevant parameters. Provide the number of functional groups detected and the number of peptides or proteins assigned to each. If functional groups were quantified, describe the quantification method in sufficient detail to ensure reproducibility.
Post-processing	List any tools or scripts used for downstream processing, such as normalization, visualization, statistical analysis, or rescoring. Include software versions and key parameters. Make any custom scripts critical to result reproduction publicly available.
*Data, metadata, and code availability*	
Data availability	All data, including but not limited to raw files, protein database (FASTA file), DIA library, primary search results, processed tables, and figure source data, should be deposited in a ProteomeXchange repository, such as PRIDE. We recommend a complete submission that includes processed identification results in a PSI format (e.g., mzIdentML) to enable linking peptides to spectra and ensure integration, visualization, and reanalysis The submission to the repository must include a table that allows understanding the linkage between samples and data files (e.g., SDRF-proteomics format).
Metadata availability	All metadata should be submitted in SDRF-Proteomics format. While the Metaproteomics Initiative is currently developing dedicated templates for different microbial environments in collaboration with HUPO-PSI, we recommend using the default SDRF-Proteomics template.
Code availability	Make analysis scripts and pipelines publicly available through a code-sharing platform, such as GitHub. For long-term access and citation, archive the code in a repository that provides a DOI, such as Zenodo. Clearly indicate the exact version used, for example by referencing a GitHub tag or commit hash. Include enough detail to reproduce results, figures, and tables.

This checklist summarizes key elements that we highly recommend to be reported in any metaproteomics study

## Conclusions and future outlook

Improving transparency and standardization in metaproteomics is essential to ensure reproducibility, comparability, and integration with other omics layers in microbiome research. The recommendations presented here reflect current community priorities and provide practical guidance for researchers, journals, and data repositories. While some practices can be adapted from related fields, others require tailored solutions to address the complexity of microbial communities and protein-level data.

Beyond individual studies, open data practices benefit the field as a whole. Publicly available datasets enable benchmarking, and foster the development of standardized reference collections. They also facilitate cross-study comparisons and improve traceability, for example through the use of USIs. To fully realize these benefits, transparent reporting, structured metadata, and comprehensive data deposition in public repositories must become the norm. Achieving this will require tools and workflows that make metadata capture and submission as streamlined as possible.

Continued coordination through community initiatives is key to sustaining progress. We invite scientists to contribute to the development and adoption of metaproteomics-specific standards, with the shared goal of building a more reproducible and interoperable field. Widespread uptake will also depend on alignment with journal and funder expectations, which can help incentivize adoption. As studies grow in size and complexity, future reporting guidelines may benefit from capturing metadata on study design elements, such as research hypotheses or intended comparisons, potentially supported through mechanisms, such as registered reports.

Structured metadata can be recorded using SDRF-Proteomics [16]. Ongoing efforts are adapting this format for metaproteomics-specific use, including microbiome-relevant metadata, such as taxonomic and ecological context. These developments aim to align SDRF-Proteomics with the MIxS framework [19], widely adopted in metagenomics. Tools, such as lesSDRF [36] support the creation and validation of SDRF-Proteomics files, helping researchers meet submission requirements more efficiently. However, not all elements of our reporting checklist (Table 1) can or should be captured in SDRF-Proteomics alone. Protocol-based metadata (for example, details on database construction, search engine settings, or protein inference strategies) are currently too complex to capture in structured formats, and are therefore better documented through narrative descriptions or semi-structured templates. In parallel, the integration of BioSamples [37] into metaproteomics submissions is being explored to support traceability and facilitate cross-omics integration. These initiatives are still in development but represent a growing momentum toward machine-readable, multi-omics metadata infrastructures.

Achieving true interoperability also requires semantic consistency. Controlled vocabularies, such as PSI-MS [38], and community-maintained OBO ontologies support consistent metadata annotation. However, many metaproteomics-specific elements, such as protein grouping strategies, peptide-to-taxon assignment methods, or database filtering parameters, are not yet formally defined in these resources. Community contributions to ontology development will therefore be required to enable machine-readable annotation of these concepts.

Ultimately, achieving reproducibility, interoperability, and long-term data reuse will require widespread adoption of the proposed reporting checklist (Table 1). While SDRF-Proteomics currently serves as the default format for proteomics submissions, ongoing developments are tailoring it for microbiome-relevant applications. Broader adoption of the checklist, along with standardized formats, public protocols, and open-source tools, will be essential to ensure reproducibility, interoperability, benchmarking, and long-term data reuse in metaproteomics and beyond.

## Data Availability

No datasets were generated or analyzed during the current study.

## Abbreviations

1D-PAGE: One dimensional polyacrylamide gel electrophoresis
CAMPI: Critical Assessment of Metaproteome Investigation
CCS: Collision cross section
FAIMS: High field asymmetric waveform ion mobility spectrometry
FAIR: Findable, Accessible, Interoperable, and Reusable
FDR: False discovery rate
GSC: Genomics Standards Consortium
HUPO-PSI: Human Proteome Organization Proteomics Standards Initiative
IHMS: International Human Microbiome Standards
iTRAQ: Isobaric Tags for Relative and Absolute Quantitation
MAGs: Metagenome-assembled genomes
MIAPE: Minimum Information About a Proteomics Experiment
MIxS: Minimum Information about any (x) Sequence
MOMSI: Multi-Omics Metadata Standards Integration
MS/MS: Tandem mass spectrometry
Protein-SIP: Protein-stable isotope probing
PSM: Peptide-spectrum match
QC: Quality control
RDA: Research Data Alliance
SDRF-Proteomics: Sample and Data Relationship Format for Proteomics
SOP: Standard operating procedure
STORMS: Strengthening The Organization and Reporting of Microbiome Studies
STREAMS: Standards for Technical Reporting in Environmental and host-Associated Microbiome Studies
TIMS: Trapped ion mobility spectrometry
TMT: Tandem mass tag
TWIMS: Traveling wave ion mobility spectrometry
USI: Universal Spectrum Identifier
